# Prevalence and Risk Factors for Delirium on Arrival at the Emergency Room by Ambulance

**DOI:** 10.7759/cureus.79663

**Published:** 2025-02-25

**Authors:** Hideaki Sakuramoto, Jumpei Onuma, Shogo Uno, Mitsuki Ikeda, Saiko Okamoto, Hidehiko Nakano, Hideki Hasimoto, Akira Ouchi

**Affiliations:** 1 Department of Critical Care and Disaster Nursing, Japanese Red Cross Kyushu International College of Nursing, Munakata, JPN; 2 Department of Nursing, Hitachi General Hospital, Hitachi, JPN; 3 Department of Emergency and Critical Care Medicine, University of Tsukuba, Tsukuba, Japan; 4 Department of Emergency and Critical Care Medicine, Hitachi General Hospital, Hitachi, JPN; 5 Critical Care, Ibaraki Christian University, Hitachi, JPN

**Keywords:** ambulance, delirium, emergency room, older adults, psychiatry

## Abstract

Introduction: Delirium is an acute and often fluctuating disturbance in attention and awareness and is one of the organ dysfunctions caused by acute illness. A previous study revealed that 8.3% of older patients in the emergency room (ER) had delirium, and dementia and older age were identified as independent risk factors for delirium in the ER. However, different ER structures (intermediate or acute care units) and disease clusters may have different prevalence rates and risk factors.

Purpose: This study aimed to identify the prevalence and risk factors for delirium on arrival at the ER.

Methods: This was an exploratory clinical trial and retrospective cohort study conducted at a tertiary care emergency center in Japan, including patients aged ≥20 years who were transported to and hospitalized at the ER between April 1, 2023, and March 31, 2024. Delirium was evaluated using the Brief Confusion Assessment Method. Multivariate analysis was performed on the 12 variables that were significantly different in the comparison of patient characteristics upon arrival at the ER.

Results: Of the 20,660 patients who visited the ER, 1,486 patients who were transported by ambulance were included in this study. The prevalence of delirium upon arrival at the ER was 20.3% (301/1486 patients, 95% confidence intervals: 18.2-22.4). Multivariable logistic regression analysis revealed a significant association between delirium on arrival at the ER and older age (odds ratio (OR) 1.02, 95% confidence interval (CI) 1.01−1.04), past history of dementia (OR 3.94, 95%CI 2.41−6.44) and psychiatric illness (OR 2.76, 95%CI 1.41−5.40), higher Glasgow Coma Scale (GCS) scores (OR 0.34, 95%CI 0.29−0.40), and requiring oxygen therapy on arrival at the ER (OR 2.09, 95%CI 1.45−3.02).

Conclusions: The prevalence of delirium on arrival at the ER was approximately 20%. Our findings suggest that older age, history of dementia and psychiatric illness, low GCS score at presentation, and the need for oxygen therapy may be risk factors for delirium symptoms at presentation.

## Introduction

Delirium is an acute and often fluctuating disturbance of attention and awareness and is one of the organ dysfunctions caused by acute illness [1]. This form of organ dysfunction commonly occurs in hospitalized, mechanically ventilated, and emergency room (ER) patients [1,2]. Delirium is associated with adverse patient outcomes, such as prolonged hospitalization [3], higher death rates [1], and accelerated cognitive decline [4,5].

Few studies have reported the incidence and prevalence of delirium in the ER. The lack of reports on the incidence and/or prevalence of delirium in the ER is largely due to the specific nature of the ER, with strong time demands on healthcare providers and the high volume of patients. It has been reported that delirium, especially hypoactive delirium, is often missed by healthcare providers without the use of tools [6,7], which poses a serious problem regarding the quality of healthcare provided.

A previous study reported that 1.6% of patients in the ER developed hyperactive delirium [8]. Another study reported that 8.3% of older patients were delirium [9]. However, these studies focused on the incidence of delirium after admission to the ER, and there are no studies of delirium prevalence on arrival at the ER by ambulance. Therefore, the prevalence of delirium on arrival at the ER by ambulance is unknown. Risk factors for delirium in the ER have not yet been identified. Generally, risk factors for delirium include older age, disease severity, respiratory support, and the presence of dementia [3,10,11]. A previous study identified dementia and hearing impairment as independent risk factors for delirium in the ER [9]. However, different ER structures (intermediate or acute care units) and disease clusters may have different prevalence rates and risk factors.

In this study, we analyzed patient data from a tertiary care emergency center in Japan. This study aimed to identify the prevalence of and risk factors for delirium on arrival at the ER.

## Materials and methods

Study design and participants

This was an exploratory clinical trial and retrospective observational study conducted at Hitachi General Hospital, a tertiary care emergency center in Japan, including emergency patients aged ≥20 years who were transported by ambulance to and hospitalized at the emergency center between April 1, 2023, and March 31, 2024. Japan's emergency medical transport service is a publicly operated, single-provider system that ensures standardized pre-hospital care and rapid patient transport to medical facilities. The exclusion criteria were as follows: (1) patients with impaired consciousness who could not undergo the Brief Confusion Assessment Method (bCAM) evaluation [1], such as those who were unarousable to verbal stimuli for all delirium assessments (patients with an eye-opening score of 1−2 on the Glasgow Coma Scale (GCS)); (2) patients with severe dementia; (3) non-Japanese patients with limited Japanese language proficiency; (4) under 20 years old; and (5) palliative care patients. This study was approved by the Institutional Research Ethics Committee of Hitachi General Hospital (reference no. 2024-28), and the need for informed consent was waived in view of the observational nature of the study. This study adhered to the principles of the Declaration of Helsinki.

Data collection

Delirium assessments were conducted in the ER as part of routine care by nurses and were evaluated immediately after the patient's arrival by ambulance. Prior to the implementation of a delirium assessment tool, there were no specific delirium assessment tools in place at the study site. Therefore, training on the use of the bCAM was conducted for all ER nurses on three separate occasions. Retrospective data collection was performed using the electronic medical records (EMRs) of all patients transported to the ER by ambulance during the study period. In addition to bCAM evaluations, data on patient demographics and vital signs at admission were collected from progress notes, EMRs, and the medical safety management system. The outcomes included the prevalence of delirium in the ER, which was assessed using the bCAM.

Instrument

Delirium was evaluated using the bCAM [12]. The bCAM is a modification of the Confusion Assessment Method for the Intensive Care Unit (CAM-ICU). Like CAM and CAM-ICU, the bCAM has four features: (1) altered mental status or a fluctuating course, (2) inattention, (3) altered level of consciousness, and (4) disorganized thinking. For a patient to meet the criteria for delirium, they must be positive for features 1 and 2 and positive for either features 3 or 4. However, patients who are severely sedated, comatose, paralyzed, or visually impaired may have difficulty following instructions, which may limit their evaluation.

Data management

The quality and integrity of the data were assessed. Missing, extreme, or implausible values were returned to local data collectors for review. Where the data remained questionable, the primary investigators made a final adjudication regarding study inclusion by mutual agreement. Missing values were omitted from the analysis.

Sample size

Based on previous studies [2,9], the prevalence of delirium is estimated to be 8-14%. To conduct a logistic multivariate analysis with adverse event occurrence as the dependent variable, at least 1,000 patients were targeted for inclusion. This sample size accounts for approximately 10 covariates such as age, dementia, psychiatric illness, vital signs, and other factors, including medical history and the occurrence of delirium in the ER.

Statistical analysis

The prevalence of delirium was estimated with 95% confidence intervals (95%CI) using the Clopper-Pearson interval. The association between delirium on arrival at the ER and risk factors was conducted using patient demographics, vital signs on admission, and treatment data on arrival at the ER. Quantitative variables are expressed as means with standard deviations, and categorical variables are summarized as counts and percentages. Comparisons between groups were conducted using t-tests or chi-squared tests. Univariate and multivariate logistic regression analyses were performed to identify factors associated with delirium upon arrival at the ER. Statistical significance was defined as p < 0.05. All statistical analyses were performed using IBM SPSS Statistics for Windows version 29.0 (IBM Corp., Armonk, NY) and R version 4.1.0 (R Foundation for Statistical Computing, Vienna, Austria).

## Results

Patient flow and characteristics

**Figure 1:**
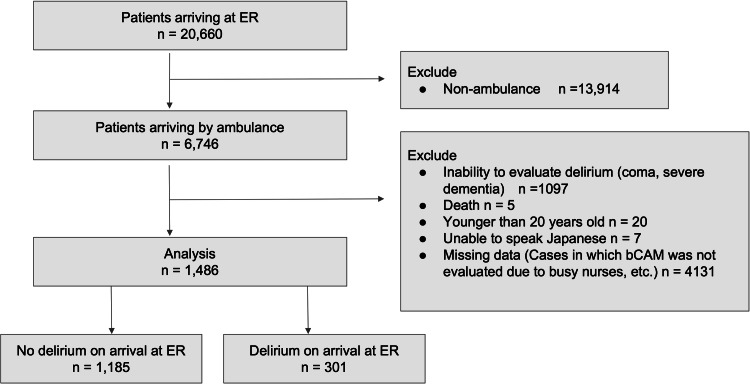
Patient recruitment flowchart Abbreviations: ER, emergency room; bCAM, the Brief Confusion Assessment Method

Of the 20,660 patients who visited the ER, 6,746 patients who were transported by ambulance were included in the study (Figure 1). Of these, 1,486 patients who were assessed using the bCAM and who did not meet the exclusion criteria were included in the analysis.

The prevalence of delirium upon arrival at the ER was 20.3% (301/1486 patients, 95%CI: 18.2-22.4). Table 1 shows the patient characteristics based on the presence or absence of delirium. The most common initial clinical diagnosis was suspected neurological disease (23.4%, 348/1486 patients). There were differences between the two groups with respect to age, sex, and history of hypertension, dementia, and psychiatric disorders. There were also differences between the two groups regarding the initial diagnosis at the ER visit, presence of suspected infection, GCS score, systolic blood pressure, respiratory rate, heart rate, body temperature, and oxygen therapy. Patients in the delirium group were also more likely to require hospitalization.

**Table 1:** Characteristics of the study population and univariable comparison with delirium Quantitative variables are expressed as means with standard deviations, and categorical variables are summarized as counts and percentages. Comparisons between groups were conducted using t-tests or chi-squared tests. Abbreviations: ER, emergency room; GCS, Glasgow Coma Scale

	No-delirium n = 1185	Delirium n = 301	p-value
Age, years, mean±SD	71.6±16.9	79.3±15.7	<0.000
Sex, female, n (%)	510 (43.0)	153 (50.8)	0.015
Past history, yes, n (%)			
Hypertension	503 (42.5)	149 (49.5)	0.028
Hyperlipidemia	123 (10.4)	28 (9.3)	0.578
Diabetes	275 (23.2)	76 (25.3)	0.456
Chronic cardiovascular disease	324 (27.3)	78 (25.9)	0.618
Chronic kidney disease	76 (6.4)	17 (5.7)	0.622
Dementia	42 (3.5)	66 (21.9)	<0.000
Psychiatric illness	60 (5.1)	26 (8.6)	<0.018
Cancer	220 (18.6)	59 (19.6)	0.681
Initial clinical diagnosis, yes, n (%)			<0.000
Respiratory	129 (10.9)	38 (12.6)	
Cardiovascular	180 (15.2)	36 (12.0)	
Neurologic	267 (22.5)	81 (26.9)	
Gastrointestinal	158 (13.3)	29 (9.6)	
Renal/genitourinary	57 (4.8)	29 (9.6)	
Metabolic/endocrine	32 (2.7)	15 (5.0)	
Hepatic/Hematologic	26 (2.2)	2 (0.7)	
Trauma/musculoskeletal	236 (19.9)	34 (11.3)	
Drug toxicity/withdrawal	22 (1.9)	16 (5.3)	
Oncology	43 (3.6)	9 (2.9)	
Other	35 (3.0)	12 (4.0)	
Suspected infection on arrival at ER, n (%)	170 (14.4)	70 (23.3)	<0.000
Vital signs on arrival at ER, mean±SD			
GCS	14.7±0.8	13.2±1.7	<0.000
Systolic blood pressure, mmHg	148.2±29.8	142.3±31.5	0.026
Respiratory rate, /min	20.3±4.8	21.1±5.1	0.013
Heart rate, /min	85.9±21.0	89.8±22.9	0.006
Body temperature,°C	36.8±0.9	36.9±1.2	0.022
SpO2, %	97.2±2.3	97.0±2.7	0.389
Oxygen treatment on arrival at ER, yes, n (%)	201 (17.0)	105 (34.9)	<0.000
Need for hospitalization, n (%)			
General ward	512 (43.2)	182 (60.5)	<0.000
Intensive care unit	51 (4.3)	29 (9.6)	

Association between delirium and GCS on arrival at the ER

**Figure 2:**
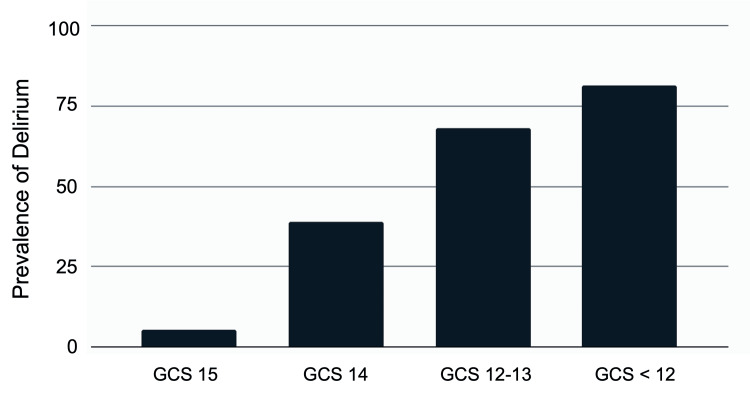
Association between delirium and GCS on arrival at the ER among 301 patients with delirium. The delirium prevalence rate is shown for each GCS score at the time of arrival at the ER. The prevalence of delirium was 5.2% at GCS 15, 39.1% at GCS 14, 68.1% at GCS 12−13, and 81.4% at GCS < 12. Abbreviations: GCS, Glasgow Coma Scale; ER, emergency room

The prevalence of delirium was 5.2% at GCS 15, 39.1% at GCS 14, 68.1% at GCS 12−13, and 81.4% at GCS < 12 (Figure 2). The prevalence of delirium decreased with higher GCS (univariable analysis odds ratio (OR) 0.30, 95% confidence interval (CI); 0.253−0.353).

Risk factors for delirium on arrival at the ER

Multivariate analysis was performed on the 12 variables that were significantly different in the comparison of patient characteristics upon arrival at the ER (Table 2). Logistic regression revealed a significant association between delirium on arrival at the ER and older age, past history of dementia and psychiatric illness, higher GCS scores, and need for oxygen therapy on arrival at the ER.

**Table 2:** Multivariate logistic regression analysis of risk factors for delirium on arrival at the ER Multivariate logistic regression analyses were performed to identify factors associated with delirium upon arrival at the ER. Abbreviations: ER, emergency room; GCS, Glasgow Coma Scale; CI, confidence interval

	Odds ratio	95% CI	p-value
Age, years	1.02	1.01-1.04	<0.000
Female	0.90	0.65-1.23	0.499
Hypertension, yes	1.15	0.83-1.60	0.404
Dementia, yes	3.94	2.41- 6.44	<0.000
Psychiatric illness, yes	2.76	1.41-5.40	0.003
Suspected infection on arrival at the ER, yes	1.14	0.73-1.77	0.559
GCS on arrival at the ER	0.34	0.29-0.40	<0.000
Systolic blood pressure on arrival at the ER, mm Hg	1.00	0.99-1.00	0.266
Respiratory rate on arrival at the ER, /min	1.01	1.00-1.04	0.770
Heart rate on arrival at the ER, /min	1.01	1.00-1.01	0.180
Body temperature on arrival at the ER, °C	1.00	1.00-1.00	0.653
Oxygen therapy on arrival at the ER, yes	2.09	1.45-3.02	<0.000

## Discussion

To our knowledge, this is the first study to investigate the prevalence and risk factors for delirium upon arrival at the ER. This study suggested that 20% of the patients evaluated by bCAM have delirium at the time of their visit to the ER of tertiary emergency center by ambulance. The results suggest that, in addition to the traditional risk factors for delirium, a low GCS score and the need for oxygen therapy on arrival at the ER are risk factors for the development of delirium. Furthermore, patients with delirium symptoms upon arrival at the ER were more likely to be hospitalized.

Compared to a previous study [9], the prevalence of delirium in this study was significantly higher than previously reported. This may be due to the fact that this study included only relatively urgent and severely ill patients transported by ambulance. A recent meta-analysis reported a higher prevalence of 31% in the intensive care unit (ICU) and other severely ill patients [13]. It cannot be ruled out that the symptom rate might have been similar to that in previous studies if patients who presented to the hospital by non-emergency means were included. Older individuals were an important risk factor for delirium in the ER, possibly due to an age-related decline in brain function, similar to previous studies [8,9,14,15]. In this study, the participants were older, with a mean age of over 70 years, which may be associated with a higher prevalence of symptoms.

In addition to the traditionally recognized risk factors for delirium [15], we found two additional risk factors for delirium in the ER: low GCS score and the need for oxygen therapy upon ER arrival. This is consistent with previous studies of patients admitted to emergency ICUs [16]; however, this study assessed patients upon arrival in the ER and was easier to screen because of the simplicity of the GCS assessment tool. Lower GCS scores are strong predictors of delirium, suggesting that brain dysfunction associated with acute illness significantly contributes to its development [1]. In addition, a history of dementia or psychiatric illness, which can affect GCS scores, has been reported as a general risk factor for delirium [17]. This factor should be highlighted, as it considerably increases the likelihood of developing delirium in patients presenting to the ER.

Significance and implications

The prevalence of delirium among patients presenting to the ER is notably high and is expected to increase as the population continues to age. Patients with delirium upon arrival at the ER exhibited a high rate of hospitalization. In hospitalized patients, the development of delirium has consistently been associated with serious adverse events and is known to significantly impact patient outcomes [1]. Consequently, early identification of patients at risk for delirium upon arrival at the ER and the implementation of prophylactic interventions may improve patient outcomes and merit further investigation. Therefore, proactive screening for delirium at the time of admission to the ER is crucial.

Strengths and limitations

To our knowledge, this study is the first to investigate the prevalence of and risk factors for delirium upon arrival at the ER. However, this study has several limitations that must be acknowledged. First, because this was a single-center retrospective study, caution should be exercised when generalizing our findings. Future multicenter studies are necessary to explore the prevalence of delirium and its associated risk factors at the time of ER admission. Second, although the study included a large sample size of over 1,000 patients, it did not include all patients transported to the ER by ambulance. Therefore, obtaining accurate prevalence estimates is challenging. To address this issue, the Clopper-Pearson method was used to estimate 95% confidence intervals for the prevalence rate, enhancing its robustness. However, caution is required when interpreting the results. Third, several patients in this study could not be evaluated with the bCAM. This limitation underscores the practical challenges of screening all patients with delirium in the ER. Although the bCAM is suitable for use in the ER, the high volume of outpatients, complex workflows, and time constraints may have contributed to this challenge. Additionally, the absence of legal regulations regarding nurse-to-patient ratios in Japanese ERs and limited staffing may have further affected the feasibility of comprehensive screening. Future research is required to develop study designs that enable more exhaustive patient assessments in this context.

## Conclusions

The prevalence of delirium upon arrival at the ER was approximately 20%. In addition, the study identified potential risk factors for delirium at presentation, including older age, history of dementia or psychiatric illness, low GCS score at presentation, and requiring oxygen therapy. The early identification of patients at risk for delirium upon arrival at the ER and the implementation of prophylactic interventions may improve patient outcomes and merit further investigation.
